# Early versus delayed penile prosthesis insertion for refractory ischemic priapism

**DOI:** 10.1080/2090598X.2022.2135290

**Published:** 2022-10-15

**Authors:** Baher Salman, Eid Elsherif, Mohamed Elgharabawy, Atef Badawy

**Affiliations:** Urology Department, Faculty of Medicine, Menofia University, Shibin Elkoom, Egypt

**Keywords:** Priapism, penile prosthesis, erectile dysfunction

## Abstract

**Objectives:**

Penile prosthesis insertion is a well-established therapeutic option in refractory ischemic priapism but there is a lack of standardization regarding the timing of surgery, the type of prosthesis (malleable or inflatable), as well as the possible complications. In this study, we retrospectively compared early versus delayed penile prosthesis insertion in patients with refractory ischemic priapism.

**Methods:**

42 male patients who presented with refractory ischemic priapism during the period between January 2019 and January 2022 were included in this study. All patients had malleable penile prosthesis insertion by four highly experienced consultants. Patients were divided into two groups based on the time of the prosthesis insertion. 23 patients had immediate insertion of the prosthesis within the first week of the onset of priapism while the remaining 19 patients had delayed prosthesis insertion three months or later after the onset of priapism. The outcome as well as the intra- and the postoperative complications were recorded.

**Results:**

Postoperative complications such as prosthesis erosion and infection were higher among the early insertion group while the delayed insertion group had higher incidence of intraoperative complications such as corporal perforation and urethral injury. The insertion of the prosthesis was much more difficult among the delayed insertion group due to fibrosis which made dilatation of the corpora very difficult. The length and the width of the penile implant were significantly higher among the early insertion group as compared to the delayed insertion group.

**Conclusions:**

Early penile prosthesis insertion for refractory ischemic priapism is a safe and effective treatment option as delayed prosthesis insertion is more difficult and challenging due to corporal fibrosis and is associated with higher complication.

## Introduction

Priapism is defined as painful prolonged erection for more than four hours which is not relieved by ejaculation, not associated with sexual stimulation, and is maintained despite orgasm [1].

Up to 95% of cases of priapism are of the refractory ischemic type and results from obstruction of the penile venous outflow and stasis of hypoxic blood within the corpus cavernosum. The progressive ischemia within the cavernosal tissue is associated with time dependent changes in the corporal metabolic environment, which leads to smooth muscle necrosis [2].

Ischemic priapism like any compartmental syndrome is a urologic emergency and should be managed as early as possible and if left untreated, will be associated with penile fibrosis, shortening and erectile dysfunction [3].

Distal shunt for treatment of ischemic priapism provides new channels for the ischemic blood which is accumulated in the corpus cavernosum, this leads to detumescence and pain relieve however future erectile dysfunction is the main side effect [4].

Although penile prosthesis insertion is a well-established therapeutic option in ischemic priapism, there is a lack of randomized studies describing the detailed use of it. The timing of surgery, the type of prosthesis (malleable or inflatable), and the possible complications are still deficient in the literature [5].

Early penile prosthesis insertion is easy, alleviates pain, provides early return to sexual activity, maintain penile length, and prevent future penile shortening due to corporal fibrosis [6]. Despite the above-mentioned advantages, immediate penile prosthesis insertion is associated with penile edema, increased risk of infection and distal perforations especially in patients who had past history of shunt procedures [7].

Delayed penile prosthesis insertion in the fibrotic corpora due to prolonged or recurrent ischemic priapism is considered as a surgical challenge with high complication rate [8].

There is no general agreement regarding the timing of penile prosthesis insertion in patients with ischemic priapism as there is no definite time after which irreversible damage of the corpus cavernosum will occur. However, most authors agree that irreversible damage of corpus cavernosum occurs within 48 h [9].

In this study, we evaluated patients with early penile prosthesis insertion compared to those with delayed prosthesis insertion after ischemic priapism regarding the success rate and the possible complications in each group.

## Materials and methods

This is a retrospective study that included 42 male patients who presented with refractory ischemic priapism from January 2019 to January 2022. The study was carried out at the urology department, Menofia university hospital and a private hospital in Menofia governorate. Four urology consultants with high experience in penile prosthesis insertion performed the procedures. Patients were divided into two groups based on the time of prosthesis insertion. 23 patients had immediate insertion of the prosthesis within the first week of the onset of priapism while the remaining 19 patients had delayed prosthesis insertion three months or later after the onset of priapism, so the fibrosis of the tissue would be settled. Those 19 patients either refused immediate insertion of the prosthesis after the onset of priapism or could not financially afford the cost of immediate prosthesis insertion. They developed erectile dysfunction later on as a consequence of prolonged ischemic priapism and came for penile prosthesis insertion.

The procedure was performed under spinal anesthesia, with prophylactic broad spectrum antibiotic (third-generation cephalosporins) before skin incision as recommended by local infection control unit in our hospital. Midline penoscrotal incision was performed after urethral catheter insertion. Dilatation with Hegar’s dilators and repeated irrigation with gentamycin diluted solution was done.

In cases with delayed prosthesis insertion, dilatation was difficult and the fibrous tissues were dissected and excised to allow for adequate dilatation of the corpora. No drain was used and the urethral catheter was removed on the first postoperative day before patients discharge. All patients were maintained at home on broad spectrum injection antibiotics (third-generation cephalosporins) for 5 days and continued on oral quinolones and Augmentins for another 10 days. Patients were instructed to call the surgeon if they noticed any complications after discharge and came for regular follow-up after 1 week, 2 weeks and then after 1 month. Sexual activity was allowed after 6 weeks unless there is complication.

The malleable penile prosthesis type was the American Medical Systems (AMS) Spectra implant (Minnetonka, MN, USA) in about 20 patients and the TUBE (Promedon) malleable implant in the remaining 22 patients, and this was dependent on the available device at the time of surgery.

Patients’ demographics, causes of priapism, risk factors for erectile dysfunctions as well as the intraoperative and the post-operative complications were recorded.

Statistical analysis was performed by IBM SPSS v 20. Student t-test was used for mean, while Chi^2^ and Fisher’s Exact Tests were used for categorical variable. P values less than 0.05 were considered significant.

## Results

Mean age of the patients was 55 ± 9.5 years for the early insertion group and 54 ± 13 years for the delayed insertion group with no statistical significant difference (Independent samples T-Test, P = 0.7). Risk factors for erectile dysfunction are shown in Table 1. The etiology of priapism is shown in Table 2.

**Table 1.** Risk factors for erectile dysfunction.

Risk factor for erectile dysfunction	Early insertion	Delayed insertion	P Value*
Diabetes mellitus	14/23 (61%)	10/19 (53%)	0.59
Hypertension	10/23 (44%)	9/19 (47%)	0.8
Smoking	15/23 (65%)	16/19 (84%)	0.16
Hyperlipidemia	12/23 (52%)	13/19 (68%)	0.28

Note: * chi square.

**Table 2.** Aetiology of priapism.

Etiology of priapism	Early insertion	Delayed insertion	P Value
Intracavernosal injection	9/23 (39%)	6/19 (32%)	0.6*
Sickle cell disease	2/23 (9%)	4/19 (21%)	0.7**
Thalassemia	2/23 (9%)	2/19 (10%)	0.7**
Idiopathic	10/23 (43%)	7/19 (37%)	0.6*

Notes: *Chi square. **Fisher’s Exact Test.

Conservative treatment of priapism in the form of aspiration and phenylephrine injection was done in 15 patients (65%) of the early insertion group and in all (19) patients of the delayed insertion group with no statistical significant difference between the two groups, (chi-square test, P = 0.005).

Distal shunt procedures were carried out in eight patients (35%) of the early insertion group (El-Ghorab in five and winter procedure in three patients) and in 13 patients (68%) of the delayed insertion group (El-Ghorab in eight and winter procedure in five patients) with no statistical significant difference between the two groups, (chi-square test, P = 0.06). The remaining patients in the early insertion group were subjected to immediate prosthesis insertion but those in the delayed insertion group were counseled about the benefit and the draw backs of the shunt procedures but they refused for fear of erectile dysfunction.

In those patients who underwent distal shunt procedures, there was no statistically significant difference between the studied groups regarding the intraoperative and the post-operative complications except intraoperative corporal perforation which was higher among the delayed insertion group due to extensive corporeal fibrosis and Postoperative penile edema which was higher among the early insertion group as shown in Table 3. The distal shunt procedures were not associated with high incidence of intraoperative corporal perforation in the early group as the shunts were of small opening and no fibrosis of tissues was present in the early group.

**Table 3.** Relationship between the distal shunt procedures and the intraoperative and the postoperative complications among the studied groups.

Type of complications	Distal shunt procedure		P value *
	Early insertion group (8)	Delayed insertion group (13)	
Intraoperative corporal perforation	0 (0%)	7 (54%)	0.018
Distal Implant erosion	2 (25%)	0 (0%)	0.133
Postoperative device infection	1 (12.5%)	0 (0%)	0.38
Postoperative wound infection	3 (37.5%)	2 (15%)	0.32
Postoperative penile edema	6 (75%)	0 (0%)	0.001

* Fisher’s Exact Test

The mean duration from the onset of priapism till insertion of prosthesis was 5 ± 1.4 days among the early insertion group and 177 ± 60 days among the delayed insertion group, (Independent samples T- Test, P 0.001).

The insertion of the prosthesis in the delayed group was more difficult and took more time due to corporal fibrosis which made dilatation of the corpora very difficult. Furthermore the inserted prosthesis length and girth were less than the early insertion group as shown in Table 4.

**Table 4.** Duration of the procedure and prosthesis dimensions.

Duration of the procedure and prosthesis dimension	Early insertion group	Delayed insertion group	P Value*
Duration of the procedure (Mean ± SD) (minutes)	102 ± 17	169 ± 22	0.000
Implant length (Mean ± SD) (cm)	22 ± 1	20.7 ± 1	0.000
Girth of the implant (Mean ± SD) (mm)	11 ± 0.5	10 ± 0.7	0.000

T-test*

The incidence of postoperative complications was higher among the early insertion group while the delayed insertion group had higher incidence of intraoperative complications as shown in Table 5.

**Table 5.** Intraoperative and postoperative complications.

Complications	Early insertion n- %	Delayed insertion n- %	P Value
Corporal perforation	1 (4%)	11 (58%)	0.001*
Urethral injury	0 (0%)	4 (21%)	0.03**
Penile edema	14/23 (60%)	0 (0%)	0.001*
Wound infection	6/23 (26%)	2 (10%)	0.2**
Distal implant erosion	2/23 (9%)	0 (0%)	0.4**
Prosthesis infection	2/23 (9%)	0 (0%)	0.4**

Notes: *Chi-square **Fisher’s Exact Test.

Distal corporal perforation was seen in the early insertion group and in seven cases from the delayed insertion group. The remaining four patients in the delayed insertion group have proximal corporeal perforation. Corporal perforation was managed by suturing with 2/0 non-absorbable sutures, and prolene mesh was used to augment the corpora in four cases in the delayed insertion group as the corporeal defect were large.

**Figure 1.**
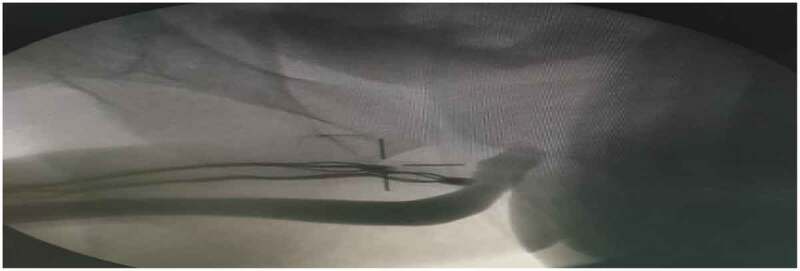
Ascending urethrogram after 3 weeks of urethral injury during prosthesis insertion.

Urethral injury was seen in the delayed group only due to extensive fibrosis and was found at the penile urethra in two cases, bulbar urethra in one case and posterior urethra in one case. Most cases were managed by suturing with absorbable sutures and urethral catheter drainage for 3 to 4 weeks and the prosthesis were inserted. Postoperative ascending urethrogram was done before catheter removal to ensure complete healing and no communication with the corpora as shown in Figure 1.

Penile edema was managed by anti-inflammatory drugs and local hot fomentation with resolution in most patients by 2 weeks with no serious complications developed. Wound infection was managed by repeated dressing and wound cleaning with changing the type of antibiotics according to the wound culture.

**Figure 2.**
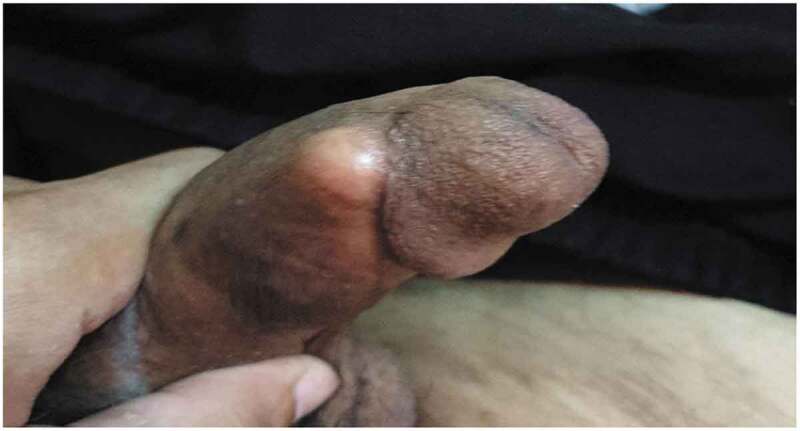
Imminent penile prosthesis perforation.

Mean follow-up period was 11 ± 5.5 months and 15 ± 4.5 months for the early and the delayed insertion group, respectively. All patients with delayed insertion who wait for 3 or more months after priapismic episodes and suffered from penile fibrosis, shortening and impotence for this long time when returned to regular sexual life after prosthesis insertion were satisfied with the prosthesis. Two patients (9%) from the early insertion group were not satisfied after prosthesis insertion due to distal implant erosion as shown in Figure 2 that was managed by decreasing the size of the implant and suturing the corpora by non-absorbable sutures but unfortunately prosthesis infection occurred that required removal of the implants.

## Discussion

Although penile prosthesis insertion is a well-established therapeutic option in ischemic priapism, the decision of penile prosthesis in acute attack of ischemic priapism is based on expert opinion, which based on small or retrospective studies, so decision-making should be shared between patient and the urologist [5].

With different types of priapism and variant etiologic factors, the goal for treating all patients of priapism is the same, which achieves detumescence, decrease pain, preserve potency power and prevent recurrent future attacks [10].

Although about one-third of cases have no specific etiology, vasoactive intracorporeal injection, psychotropic medications, sickle cell anemia, thalassemia, and corporeal local infiltration by pelvic malignancies are the most common causes [2].

Conservative treatment and aspiration with or without injection of phenylephrine is usually effective in early stages; however, it is mostly useless after 48 h. Surgical intervention in the form of penile shunt surgery and penile implant insertion for refractory or delayed ischaemic priapism should be considered when other conservative management options fail [11].

There are two main types of shunt procedures, distal (such as winter and Alghorab operations) and proximal (such as corporal-spongiosum and corporal-saphenous shunt). The distal type should be tried first due to lower complication rate; however, the proximal type is more effective in achieving detumescence but carry the risk for erectile dysfunction more than the distal types (50% versus 25%) [12].

The role of percutaneous shunt procedures is still questionable in preserving cavernosal smooth muscle viability, as it usually follow failed conservative treatment and cavernosal injection which mostly indicates smooth muscles necrosis [13].

Infection is the most common complication after immediate penile prosthesis insertion, especially in patient who underwent aspiration, injection or shunt surgeries. This may be due to bacterial penetration through the skin to the sterile compartment and cavernosal edema which may prevent antibiotic penetration to the cavernosal tissues [14].

Penile prosthesis insertion after well-established corporeal fibrosis can carry big challenges. Difficult corporeal dilatation with hegars dilators may be replaced by cavernotomes to overlap difficult dilation which is named corporeal drilling. This difficult situation may be complicated with urethral injury, lateral or distal perforation or cylinder crossover [13].

Extensive corporeal fibrosis and difficult dilatation obligate the surgeon to deliberately downsize the penile prosthesis size to accommodate the corporeal compartment. The decreased penile length is associated with lower patient satisfaction [14].

In our study, we have 2 out of 23 (9%) that required revision surgery due to prosthesis migration (Figure 2). All of them developed post-operative infection. This high rate of infection may be due to shunt procedures with bacterial invasion through penile skin and penile edema which prevent proper antibiotic penetration.

In the study done by Ralph et al [15], there were 3 out of 50 patients (6%) required revision surgery due to potential weakness at the corporeal tips at the site of previous distal perforation.

In contrast to our study, Salem et al [16] reported zero incidence of infection and distal erosion, this may be due to Salem et al novel technique of non-absorbable sling suture for fixing the prosthesis in place which prevent its migration.

Although the exact time for complete fibrosis after acute priapism attack is still not known, Sedigh et al [9] described easy dilatation after one week of priapism. In our study the dilatation was easy within 5 days of the attack.

Although both malleable and inflatable penile prosthesis have been tried in acute attack of priapism [17], we selected the malleable type in our study as the malleable type act as a stretching device which prevent penile shortening with possible future exchange with inflatable one after at least three months. Also in our institute, the health insurance system does not cover the prosthesis surgery and most patients choose the cheapest one which is the malleable.

Zacharakis et al [14] supported the malleable penile prosthesis insertion for early penile length preservation, no need for early cycling of the inflatable device and easy future exchange to inflatable one. However, Sedigh et al [9] recommended inflatable penile prosthesis as a good first line therapy if we used antibiotic-coated prosthesis and cylinder oversizing. In our study, patients refused to change the prosthesis type because of financial reasons, and they were satisfied with the malleable prosthesis.

This study had some limitation as the sample size was small as most cases of priapism usually resolve with the conservative treatment. Additionally, the expensive cost of different types of the prosthesis added more cost to the operation in a country with no medical insurance for all patients that may hinder some patients with refractory ischemic priapism from immediately insert the prosthesis. We used only two types of devices so further multi-centers studies with different devices types are recommended.

## Conclusion

Early penile prosthesis insertion after failure of conservative treatment for refractory ischemic priapism is a safe and effective treatment option instead of shunt procedure as shunt procedure is associated with higher complication, higher failure rate and delayed regaining sexual activity. Furthermore delayed prosthesis insertion is more difficult and challenging due to corporeal fibrosis and is associated with higher complication and decreasing the size of the inserted prosthesis.

## References

[1] Sadeghi-Nejad H, Seftel AD. The etiology, diagnosis, and treatment of priapism: review of the American Foundation for Urologic Disease Consensus Panel Report. Curr Urol Rep. 2002;3(6):492–498. 12425873 10.1007/s11934-002-0103-7

[2] Broderick GA, Kadioglu A, Bivalaqua TJ, et al. Priapism: pathogenesis, epidemiology and management. J Sex Med. 2010;7:476–500. 20092449 10.1111/j.1743-6109.2009.01625.x

[3] El-Bahnasawy MS, Dawood A, Farouk A. Low-flow priapism: risk factors for erectile dysfunction. BJU Int. 2002;89(3):285–290. 11856112 10.1046/j.1464-4096.2001.01510.x

[4] Spycher MA, Hauri D. The ultrastructure of the erectile tissue in priapism. J Urol. 1986;135(1):142–147. 3941454 10.1016/s0022-5347(17)45549-2

[5] Reddy AG. Role of Penile Prosthesis in Priapism: a Review. World J Mens Health. 2018;36(1):4–14. 29299902 10.5534/wjmh.17040 PMC5756805

[6] Minervini A, Ralph DJ, Pryor JP. Outcome of penile prosthesis implantation for treating erectile dysfunction: experience with 504 procedures. BJU Int. 2006;97(1):129–133. 16336342 10.1111/j.1464-410X.2005.05907.x

[7] Ralph DJ, Borley NC, Allen C, Kirkham A, Freeman A, Minhas S, Muneer A, et al. The use of high-resolution magnetic resonance imaging in the management of patients presenting with priapism. BJU Int. 2010;106(11):1714–1718. 20438564 10.1111/j.1464-410X.2010.09368.x

[8] Wilson SK, Delk JR, Mulcahy JJ, et al. Upsizing of inflatable penile implant cylinders in patients with corporal fibrosis. J Sex Med. 2006;3:736. 16839331 10.1111/j.1743-6109.2006.00263.x

[9] Sedigh O, Rolle L, Negro CL, Ceruti C, et al. Early insertion of inflatable prosthesis for intractable ischemic priapism: our experience and review of the literature. Int J Impot Res. 2011;23(4):158–164. 21654814 10.1038/ijir.2011.23

[10] Montague DK, Jarow J, Broderick GA, Dmochowski RR, et al. American Urological Association guideline on the management of priapism. J Urol. 2003;170(4 Pt 1):1318–1324. 14501756 10.1097/01.ju.0000087608.07371.ca

[11] EAU gudeline 2022. https://uroweb.org/guidelines/sexual-and-reproductive-health/chapter/priapism

[12] Ridgley J, Raison N, Sheikh MI, Dasgupta P, Khan MS, et al. Ischaemic priapism: a clinical review. Turk J Urol. 2017;43(1):1–8. 28270944 10.5152/tud.2017.59458 PMC5330261

[13] Capece M, Gillo A, Cocci A, Garaffa G, Timpano M, Falcone M, et al. Management of refractory ischemic priapism: current perspectives. Res Rep Urol. 2017;9:179–185. 10.2147/RRU.S128003 PMC5587151 28920056

[14] Zacharakis E, Garaffa G, Raheem AA, Christopher AN, et al. Penile prosthesis insertion in patients with refractory ischaemic priapism: early vs delayed implantation. BJU Int. 2014;114(4):576–581. 25383397 10.1111/bju.12686

[15] Ralph DJ, Garaffa G, Muneer A, Freeman A, Rees R, Christopher AN, et al. The immediate insertion of a penile prosthesis for acute ischaemic priapism. Eur Urol. 2009;56(6):1033–1038. 18930579 10.1016/j.eururo.2008.09.044

[16] Salem EA, El Aasser O. Management of ischemic priapism by penile prosthesis insertion: prevention of distal erosion. J Urol. 2010;183(6):2300–2303. 20400140 10.1016/j.juro.2010.02.014

[17] Kabalin JN. Corporeal fibrosis as a result of priapism prohibiting function of self-contained inflatable penile prosthesis. Urology. 1994;43(3):401–403. 8134999 10.1016/0090-4295(94)90090-6

